# Shock Waves Enhance Expression of Glycosphingolipid Tumor Antigen on Renal Cell Carcinoma: Dynamics of Physically Unmasking Hidden Intracellular Markers Independent of Gene-Signaling Pathways

**DOI:** 10.3390/biomedicines10030545

**Published:** 2022-02-24

**Authors:** Nushin Hosano, Zahra Moosavi-Nejad, Makoto Satoh, Hamid Hosano

**Affiliations:** 1 Department of Biomaterials and Bioelectrics, Institute of Industrial Nanomaterials, Kumamoto University, Kumamoto 860-8555, Japan; nushin@kumamoto-u.ac.jp; 2 Department of Biotechnology, Alzahra University, Tehran 1993893973, Iran; z.moosavinejad@alzahra.ac.ir; 3 Department of Urology, Tohoku Medical and Pharmaceutical University, Sendai 983-8536, Japan; ms.hifu@tohoku-mpu.ac.jp

**Keywords:** cancer immunotherapy, enhanced tumor-associated antigens, human renal cell carcinoma, shock waves, glycosphingolipid

## Abstract

Antigens associated with tumors have proven valuable in cancer immunotherapy. Their insufficient expression in the majority of tumors, however, limits their potential value as therapeutic markers. Aiming for a noninvasive approach applicable in clinical practice, we investigated the possibility of using focused shock waves to induce membrane expression of hidden intracellular tumor markers. Here, we studied the in vitro effect of a thousand focused shock waves at 16 MPa overpressure on the membrane expression of a cytosolic glycosphingolipid, monosialosyl-galactosyl-globoside (MSGG). Double-staining flow cytometry with propidium-iodide and monoclonal antibody RM1 revealed an immediate increase in MSGG expression on renal carcinoma cells (18% ± 0.5%) that reached its peak value (20.73% ± 0.4%) within one hour after the shock waves. The results of immunoelectron microscopy confirmed the incorporation of MSGG into newly formed cytosolic vesicles and their integration with the cell membrane. Based on the enzymatic nature of MSGG production that is not controlled directly by genes, the immediate upregulation of MSGG membrane expression implies that a chain of mechanochemical events affecting subcellular structures are responsible for the shock-wave-induced antigenic modification. Physically unmasking hidden tumor antigens and enhancing their expression by focused shock waves presents a potential noninvasive method of boosting tumor immunogenicity as a theranostic strategy in cancer immunotherapy.

## 1. Introduction

Immunotherapy is an emerging complex field of cancer treatment that relies on the immune system of the patient to fight the disease [1]. In recent years, immunotherapeutic approaches have significantly improved the efficacy of cancer treatments for both early- and late-stage patients. Several types of immunotherapeutic strategies are currently available in clinical settings, including immune checkpoint inhibitors (ICIs), T-cell therapies, monoclonal antibodies, and cancer vaccines. Immune checkpoint inhibitors (ICIs) are a class of drugs that boost immunity by blocking the inhibitory pathways that malignant tumors use to evade the immune system [2]. Alternatively, adoptive T-cell therapy involves enhancing the ability of the immune system to directly attack tumors by increasing the number of T cells and genetically developing T cells capable of recognizing and destroying tumor cells [3]. In addition, monoclonal antibodies and therapeutic cancer vaccines specifically target distinct tumor markers derived from tumor cells [4]. Through these methods, selective and personalized antitumor effects can be achieved, thus improving treatment efficacy and limiting adverse side effects of traditional cancer therapies. In spite of significant advances in this field and its immense potential for cancer prevention and treatment, immunotherapy remains the treatment of choice for only a few types of cancers that are compatible with these methods. Studies have found that the ability of tumor cells to evade detection by the immune surveillance system is one of the leading causes of poor clinical outcome. In addition to this factor, clinical efficacy of immunotherapeutic approaches can be compromised further due to the heterogeneity of tumor cell populations in terms of their immunogenicity and their microenvironment, especially in the case of advanced cancer [5].

Studies conducted on cancer resistance to immunotherapy have shown that tumors have the ability to change their immunogenicity at the cellular level through a variety of techniques, including reduced expression of antigenic markers, decreased binding affinity for specific tumor antigens, loss of or downregulation of tumor antigens, and reduced access by monoclonal antibodies to specific tumor antigens [6]. The immune system of these patients, while responding positively to the treatment, may still be compromised at any point during the treatment if cancer cells with lower immunogenicity escape immune surveillance. Collectively, these findings provide compelling evidence that the ability of the immune surveillance system to access tumor antigens during immunotherapy is an important factor that contributes to robust mobilization of the immune system against cancerous cells.

Renal cell carcinoma (RCC) is an example of a highly metastatic tumor with about 50% possibility of distant metastases at the time of diagnosis. Despite promising results from several potential vaccines developed for RCC, tumors have shown resistance and recurrences in clinics [7]. As part of the search for potential tumor markers suitable for an immunotherapeutic approach, studies have identified high levels of tumor-associated glycosphingolipid (GSL) antigens in RCC tumor cells [8,9].

Tumor-associated GSL antigens have long been used to develop commercial diagnostic methods, owing to their ability to stimulate the immune system. Recent studies indicate that tumor-associated GSLs may also serve as potential targets for both passive and active immunotherapeutic strategies [10,11]. Furthermore, these tumor markers have also been considered as potential targets for cytotoxic T lymphocytes (CTLs) in ex vivo vaccine development [12]. The increasing number of successful preclinical and experimental applications using tumor-associated GSLs has encouraged an optimistic view of these markers as therapeutic targets. Nevertheless, tumor-associated GSLs must be expressed above a certain threshold level in the cell membranes of tumors in order to be immunogenic [8]. It is therefore imperative to enhance the expression of these markers on tumor cells, particularly those hidden GSLs with the potential to stimulate the immune system, in order to trigger an effective immune response in patients.

As part of a successful treatment plan, it is critical to ensure that available immunogenic markers are enhanced in order to induce an immunogenic response. It is possible to achieve this goal by exposing an abundance of hidden intracellular antigens within tumor cells and by increasing their expression in order to facilitate the infiltration of immune cells, primarily T cells, into the tumor microenvironment. Several recent preclinical studies have indicated that using immunotherapy and radiotherapy together can increase the effectiveness of treatment in a synergistic manner [13]. Radiation-induced cell damage is believed to expose tumor antigens and make tumor cells visible to the immune system. Given the extent of side effects associated with radiotherapy, it is nevertheless desirable to identify alternative approaches capable of transferring such destructive energy into the body in a targeted but non-invasive manner so that it can destroy the tumor cells with no or few side effects.

As a long-standing clinical practice, applications of shock waves are known for their ability to cause a variety of bioeffects within the body, ranging from killing cells to modifying their subcellular structures [14]. Over the past several decades, extensive research has expanded our knowledge of the mechanisms that allow shock waves to exert mechanobiological influences in experimental and clinical models. Furthermore, intracellular antigens within the targeted tissues can be exposed by shock waves. Reports of systemic immune reactions following extracorporeal shock wave lithotripsy (ESWL) support this hypothesis that the reactions may be caused by exposure of the immune system to intracellular antigens [15]. Further evidence can be found in a report of mesangial glomerulopathy in both treated and control kidneys of pigs after only one kidney had been exposed to focused shock waves. The immune responses in the untreated control kidneys suggested that the proliferative factors were circulating in the treated animals with the possibility of an immune-complex mechanism for the mesangial lesion [16]. In addition to focused shock waves, high-intensity focused ultrasound (HIFU) is also known for its thermal and mechanical destruction properties in order to produce antitumor effects. However, mechanically induced immune-modulating results via shock waves were shown to be more effective than those resulting from thermal strategies, such as HIFU [17]. Although it is widely believed that the release of intracellular markers from damaged cells following ESWL triggers autoimmune responses, this may not always be the case when the autoimmune reaction is caused by the abnormal presence of cytoplasmic antigenic precursors in the cell membrane of the treated cells [18]. Instead, this raises the possibility that hidden cytoplasmic markers are expressed on the surface of shock-wave-treated cells as a result of their incorporation into the cell membrane.

In this study, we investigated the in vitro impact of focused shock waves on membrane expression of MSGG, a tumor-associated GSL antigen located in the cytoplasm of renal cell carcinoma (TOS-1). In addition, underlying biophysical mechanisms governing mechanically enhanced expression of tumor-associated GSL markers on the surface of tumor cells are revealed and discussed.

Here, we explore the possibility of physically increasing the surface expression of normally hidden intracellular GSL tumor markers in the surviving cells treated with shock waves in order to enhance their visibility for the immune system and thereby improve the likelihood of their detection by immune recognition. Results of this study may provide a basis for potential applications of this non-invasive method in clinical settings for diagnostic and therapeutic purposes, in combination with immunotherapy.

## 2. Materials and Methods

### 2.1. Cell Line

An established human renal carcinoma cell line (TOS-1) derived from a metastatic tissue was cultured in modified Eagle’s medium (MEM) supplemented with 10% fetal bovine serum (FBS), 1% penicillin/streptomycin and 1% L-glutamine (all from GIBCO BRL, Grand Island, NY, USA), in a humidified atmosphere of 5% CO_2_ at 37 °C [9].

### 2.2. Shock Wave Generator

**Figure 1:**
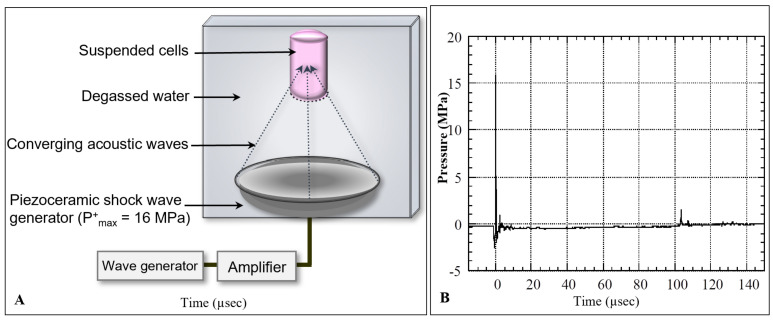
Schematic diagram of the shock wave experimental setup. (**A**) Suspended human renal carcinoma cells (TOS-1) were located in the focal area of a piezoceramic generator placed in a degassed water bath at 37 °C. Cells were exposed to shock waves at 0.2-s intervals. (**B**) Pressure history of a shock wave generated in the focal area at a voltage discharge of 1.5 kV with maximum peak pressure of P^+^_max_ = 16 MPa.

In this study, we used an experimental shock wave generator consisting of a partial spherical piezoceramic dish (125 mm inner diameter and 16 mm depth) with a 5 Hz pulse-repetition frequency at a voltage discharge of 1.5 kV, producing 16 MPa peak pressure in the focal region. The generator was placed at the bottom of a water bath (20 cm × 20 cm × 23 cm) filled with degassed water at 30 °C. A schematic diagram of the experimental set-up is shown in Figure 1A.

The pressure profile of the incident shock wave was measured by a needle hydrophone with a 0.5 mm diameter sensitive area and 50 ns rise time (Muller-Platte Needle Probe, Oberursel, Germany). The pressure history of the shock wave at the focal point with a peak positive pressure of P^+^_max_ = 16 MPa is shown in Figure 1B.

The pressure profile consisted of an earlier negative pressure of P_−_ = −2.5 MPa caused by shrinkage of the piezoceramic element, followed by a positive shock wave of 16 MPa pressure, a 0.6 µs pulse duration, and a 0.04 mJ/mm^2^ positive-energy flux density. The shock wave was followed by a tensile wave of P_−_ = −2 MPa negative pressure, 1.8 µs duration, and 0.002 mJ/mm^2^ negative-energy flux density. The tensile wave behind the shock wave was induced by the expansion wave at the opening of the piezoceramic dish, which was intensified by the wave convergence, and caused cavitation in the focal area [19]. The collapse of the cavitation bubble induced a positive pressure pulse of 1.5 MPa at 104 µs. The shock wave pressures were highly reproducible (±0.05% of peak pressure), therefore the cells were exposed to the same wave profile during each exposure. The distribution of shock wave pressure around the focus was 2.4 mm in diameter and 13 mm in axial direction (−6 dB), with an oval-shaped focal volume of 39 µL [14].

### 2.3. Shock Wave Treatment

TOS-1 cells cultured for 4 days to reach a subconfluent state were briefly exposed to 0.02% EDTA solution (GIBCO BRL, Grand Island, NY, USA), washed with phosphate-buffered saline (PBS) and suspended in the medium to a final concentration of 1 × 10^6^ cells/mL. For shock wave treatment, 2 mL of the cell suspension was transferred into a polyethylene tube (13 mm inner diameter). The bottom of the tube was cut and sealed with a thin 25 µm polyethylene film with negligible attenuation so as to achieve uniform shock wave propagation with minimal reflections (the thin film had almost the same acoustic impedance as the medium). The tube was positioned in the focal area of the shock wave. Cells were exposed to 200, 400, 600, 800 or 1000 shock waves (+SW) with 5 Hz repetition frequency at 16 MPa. Sham control cells (−SW) were treated in the same way, except for the shock wave exposures.

### 2.4. Cell Viability

A trypan blue exclusion test was used to measure cell viability following different numbers of shock wave exposures. Cytotoxicity of the shock waves was measured in the presence of 10% trypan blue immediately, 1, 2 and 3 h after the treatment.

### 2.5. Flowcytometry

Expression levels of MSGG antigen were measured on the surface of viable TOS-1 cells after the shock wave treatments using double-staining flowcytometry with FITC-labeled primary monoclonal antibody (mAb-RM1) and propidium iodide (PI) [9]. In brief, 500 µL of the cell samples was incubated with 50 µL mAb-RM1 for one hour at 4 °C, washed twice with PBS washing buffer (1%BSA/PBS + 0.05%NaN_3_), treated with 50 µL fluorescein (FITC)-conjugated anti-mouse IgG + IgM (Sigma Chemical Co., Osaka, Japan) for one hour on ice in the dark, washed twice with PBS, incubated with 20 µL PI (Sigma Chemical Co., Osaka, Japan) for 30 min, suspended in 300 µL isotone solution (Becton Dickinson, Sunnyvale, CA, USA) and finally measured by flow cytometer FACScan (Becton Dickinson, San Jose, CA, USA). Autofluorescence signals measured in a group of suspended cells in PBS, without labeling them with any antibody, were subtracted from the final results. Data were analyzed with CellQuest ^TM^ Software (Becton Dickinson, San Jose, CA, USA) with a minimum of 10,000 events per sample. Sham-exposed samples were processed in the same way, except for the shock waves. For the negative control, samples were prepared as described above, without the first monoclonal antibody.

In addition, treated cells were kept in 10% FSC/MEM, and MSGG expression was measured over time after 1, 2, 3 and 4 h following the shock wave exposures. Results were compared with the data obtained immediately after the shock waves.

### 2.6. Scanning Electron Microscopy

Shock-wave-treated cells were immediately fixed in 2.5% glutaraldehyde plus 2% paraformaldehyde for 2 h and then post-fixed in 1% osmium-tetroxide solution for 1 h. After dehydration in an ascending ethanol series, cells were dried with liquid CO_2_ with a critical point dryer (Hitachi, Tokyo, Japan) and sputtered with platinum-palladium (Pt-Pd). The morphology of the shock-wave-treated cells was compared with the control under a LEM1200EX scanning electron microscope (JEOL, Tokyo, Japan).

### 2.7. Immunoelectron Microscopy

An indirect pre-embedding immunoperoxidase technique was used for subcellular localization of the GSL antigens [20]. In brief, cell pellets were fixed in 4% paraformaldehyde/0.05 M phosphate buffer for 2 h. After a serial washing in 10%, 15% and 20% sucrose/PBS, cell pellets were embedded in OCT compound, frozen in dry ice-ethanol and stored at −80 °C. Frozen sections of the cell pellet (5 µm thick) mounted on ovalbumin-coated glass slides were immersed in 1% BSA/PBS and incubated with the first mAb-RM1 for 12 h at 4 °C, followed by overnight incubation with peroxidase-conjugated F(ab’)2 fragments of anti-mouse IgM diluted at 1:100 with PBS containing 10% normal human serum as the second antibody (Histofine Kit SAB-PO; Nichirei Co, Tokyo, Japan). Diaminobenzidine DAB (Dojin, Kumamoto, Japan) was used as chromogen at a concentration of 30 mg/100 mL Tris-HCL buffer with 0.006% H_2_O_2_. To minimize the endogenous peroxidase activity, 65 mg/100 mL sodium azide was added to the DAB. The reaction with DAB was later visualized by incubating the samples with 1% osmium tetroxide for 20 min. After dehydration in ethanol, ultra-thin sections embedded in Epon were coated with lead citrate and observed with a LEM1200EX scanning electron microscope (JEOL, Tokyo, Japan).

### 2.8. Statistical Analysis

Data were analyzed using unpaired *t* test, including Welch’s correction. Data represent mean ± SD of three independent experiments. Results were considered to be significant when the corrected *p*-value was less than 0.05, indicated as *p* < 0.05 in the figure legends.

## 3. Results

### 3.1. Effect of Shock Waves on Cell Viability

**Figure 2:**
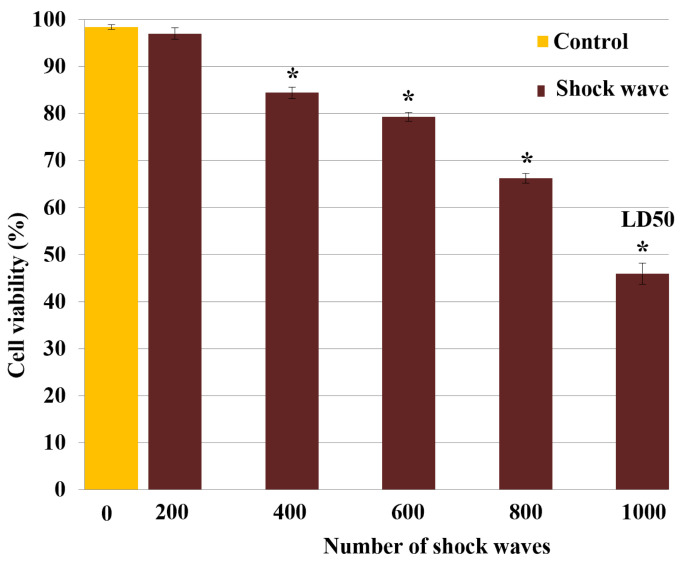
Shock wave cytotoxicity for TOS-1 renal cell carcinoma. The cell viability was measured by trypan blue exclusion test after exposure to different shock wave (SW) numbers of 200, 400, 600, 800 or 1000. A lethal dose of 50% (LD_50_) was obtained with 1000 SW exposures. Each data point represents mean ± SD (*n* = 3), * *p* < 0.05, compared with the control.

Measured TOS-1 cell viability versus the shock wave numbers based on TB exclusion test is shown in Figure 2. The cell viability after treatment with 200, 400, 600, 800 or 1000 shock waves dropped to 96.9% ± 0.9% (−2%), 87.3% ± 0.2% (−11.6%), 77% ± 0.4% (−21.9%), 68.1% ± 0.9% (−30.8%) and 49.7% ± 1.9% (−49.2%), respectively, compared with 98.9% ± 0.1% viability in the control group. On the basis of these results, 1000 focused underwater shock waves at a peak pressure of 16 MPa was selected as the standard exposure dose for the remaining shock wave treatments, which resulted in a 50% loss of cell viability in treated cells (LD_50_).

### 3.2. Effect of Shock Waves on Particle Displacement and Temperature

In this experimental setup, the volume of the shock wave focal extension (39 µL) was 2% of the total cell volume (2 mL). Since cells in the suspension were free to move with the shock-wave-induced microstreaming, during 200, 400, 600, 800 or 1000 exposures, the suspended cells experienced, on average, 4, 8, 12, 16 or 20 shock waves, respectively, with the focal pressure of 16 MPa.

The total energy applied to the cell suspension by 1000 shock wave exposures was about 8.5 J. Considering the 2 mL volume and the specific heat capacity of the suspension, a maximum increase of 1.0 °C in bulk temperature was calculated. Accordingly, there was no notable rise in temperature in the samples after 1000 shock waves.

Based on the shock Hugoniot and Tait equation of state, the particle velocity behind the shock wave was calculated to be 10.5, 7.9 and 2.7 m/s at the focus (16 MPa), focal extension (average 12 MPa), and out-of-focus zone (average 4 MPa), respectively [21,22]. Considering the shock wave pulse duration [14,23], the shock wave microstreaming resulted in an average particle displacement of 2.5, 1.9 and 0.6 µm in the above-mentioned regions, respectively. Collectively, these results indicate that shock-wave-induced stresses (impulse/microstreaming) were the prevailing physical mechanisms, whereas thermal effect was negligible.

### 3.3. Effect of Shock Waves on Membrane Expression of MSGG Antigen

#### 3.3.1. Flowcytometric Measurement

**Figure 3:**
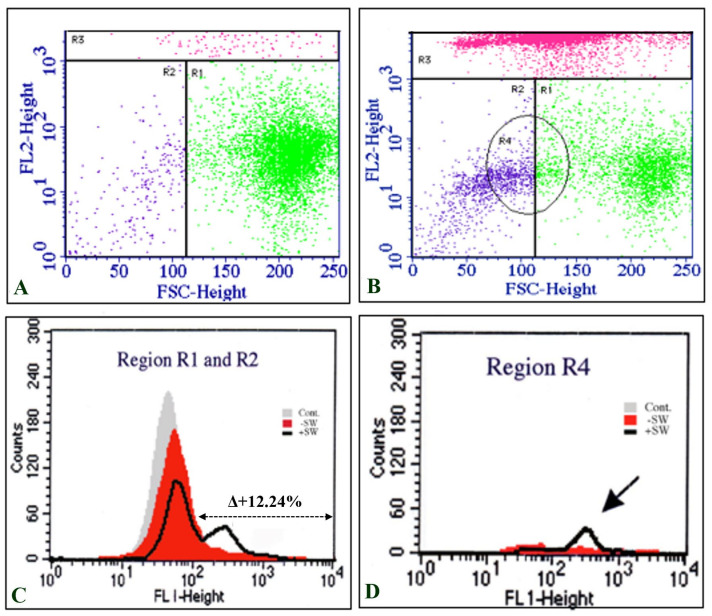
Double-staining flowcytometric analysis of TOS-1 cells. (**A**) Dot plot of the sham control cell population stained with propidium iodide (PI) (FL1-Hight). (**B**) Dot plot of the shock-wave-treated cells stained with PI. Gated R3 region represents high intensity of PI in dead cells. (**C**) Intensity of MSGG antigen stained with mAb-RM1 (FL1-Hight) on the live cells located in the area of R1 and R2 (low intensity of PI). (**D**) The majority of cells with enhanced expression of MSGG are located in R4, which represents the smaller cells (FSC-Height) in the cell population after the shock waves.

Dot plots of the total cell population in the control (Figure 3A) and shock-wave-treated cells (Figure 3B) were divided into PI positive (R3) and PI negative (R1 and R2) groups based on PI intensity (FL2-Hight). The expression level of MSGG was then measured on the surfaces of the viable cells, shown as FL1-Hight (Figure 3C–D), thereby avoiding the possibility of positive results from cross reaction with the dead cells.

Interestingly, the majority of cells with enhanced expression of MSGG were located in a subpopulation with smaller sizes than the control cells, gated in region R4 (Figure 3D).

**Figure 4:**
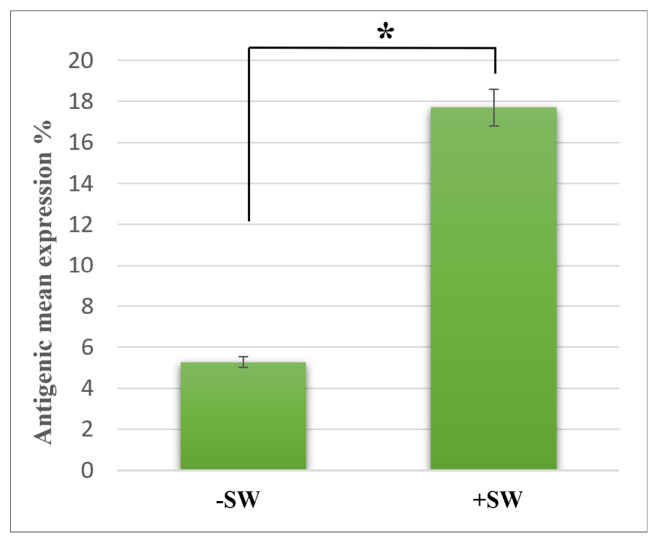
Expression of MSGG on TOS-1 renal cell carcinoma. The expression of MSGG antigen on the surface of viable TOS-1 cells was measured using double-staining flowcytometry with propidium iodide (PI) and monoclonal antibody RM1. Immediately after 1000 shock waves with 0.2-s intervals at 16 MPa overpressure, shock-wave-treated cells (+SW) showed a 12.24% increase in expression of MSGG when compared to the sham control (−SW). Each data point represents mean ± SD (*n* = 3), * *p* < 0.05, compared with the control.

Although the sham control cells (−SW) showed only 5.5% ± 0.85% expression of MSGG in the cell membrane of TOS-1 cells, a three-fold increase in the antigenic expression (18% ± 0.5%) was found immediately after shock wave exposure on the viable cells. Figure 4 shows measured membrane expression of MSGG only on the surfaces of the viable cells in the shock-wave-treated samples (+SW) compared to the sham control group (−SW).

**Figure 5:**
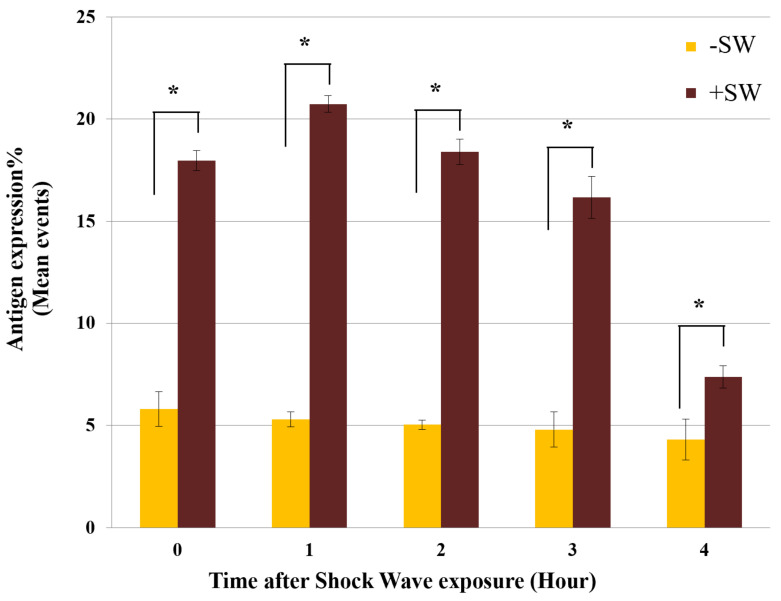
Time-course analysis of MSGG expression. Expression of MSGG on shock-wave-treated TOS-1 cells (+SW) compared with the sham controls (−SW). Each data point represents mean ± SD (*n* = 3), * *p* < 0.05, compared with their sham control at each time group.

In addition, analyzing the stability of enhanced antigenic expression over time revealed that the expression of MSGG on the shock-wave-treated cells further increased and reached its peak value of 20.73% ± 0.4% one hour after the treatment (Figure 5). After four hours, the antigen expression level was still higher than the control level but was gradually returning to normal.

#### 3.3.2. Immunoelectron Microscopic Analysis

**Figure 6:**
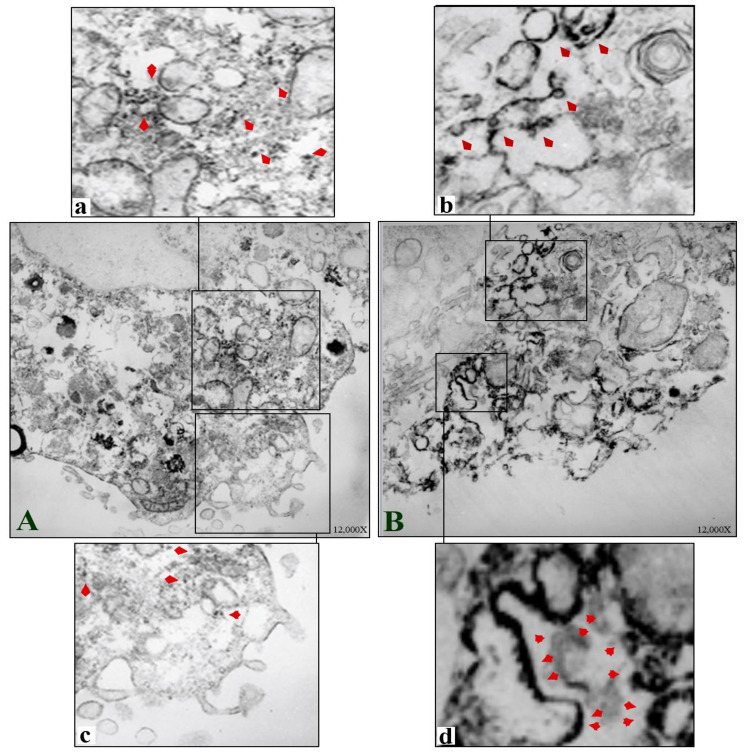
Immunoelectron microscopic images of TOS-1 renal cell carcinoma immunostained for MSGG antigen with mAb-RM1. (**A**) Control TOS-1 cell. (**a**) Cytosolic MSGG antigens non-uniformly scattered with few clusters in a control cell (arrowheads). (**c**) Enlarged view of the control cell with almost negative MSGG membrane expression. (**B**) Shock-wave-treated cell shows immediate integration of MSGG in the membranous structures. (**b**) Incorporation of MSGG antigen in cytoplasmic vesicles following the shock waves. (**d**) Strong MSGG staining of the cell membrane in the treated cells.

The ultrastructure of TOS-1 cells showed characteristics of their renal cancer origin and epithelial nature, with the cell cytoplasm containing abundant cytoplasmic lipid vesicles [9]. An immunoelectron microscopic image of a control TOS-1 cell (Figure 6A) and an enlarged image of the cell cytoplasm (Figure 6a) show MSGG antigens as electron-dense deposits in the cytoplasm, non-uniformly scattered, with a few clustering in the cytoplasm and not confined to any cellular compartment. MSGG antigens were almost absent from the cell membrane in the control cells (Figure 6c). However, an immediate shift in expression pattern of MSGG was found after the shock waves (Figure 6B). MSGG antigens in the treated cells were found mostly located in membranous structures, both in the cytoplasm (Figure 6b) and the cell membrane (Figure 6d).

**Figure 7:**
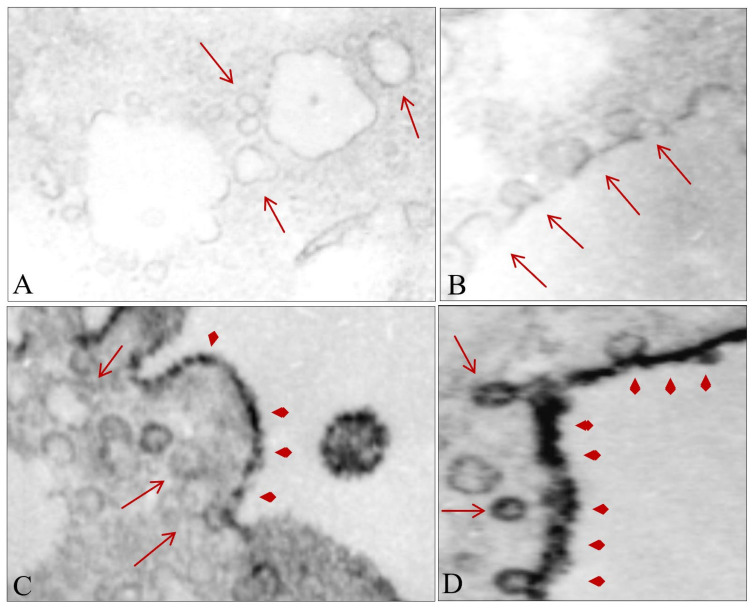
Effect of shock waves on cellular substructure. (**A**) Formation of new cytosolic vesicles after shock waves (arrows). (**B**) Growth of the cytosolic vesicles after incorporation of the smaller vesicles. (**C**) Growth in size and number of the newly formed cytosolic vesicles near the cell membrane. (**D**) Fusion of vesicles containing MSGG (arrowheads) with the cell membrane. Original magnifications, 12,000×.

In addition, the treated cells exhibited formation of 50–100 nm cytoplasmic vesicle-type structures immediately after the shock waves (Figure 7A) that grew in size and number after smaller vesicles joined them (Figure 7B). The vesicles containing a higher level of MSGG were mostly located in close proximity to the cell membrane (Figure 7C), joining the cell membrane in the treated cells (Figure 7D). With the incorporation of the antigens in the membranous structures, the cytoplasmic area of the treated cells lost their staining intensity in the immunoelectron microscopic images (Figure 7A,B). Instead, the shift in membrane expression of MSGG increased the antigen-staining intensity of the cell membrane in the treated cells (Figure 7C,D).

### 3.4. Morphological Analysis

**Figure 8:**
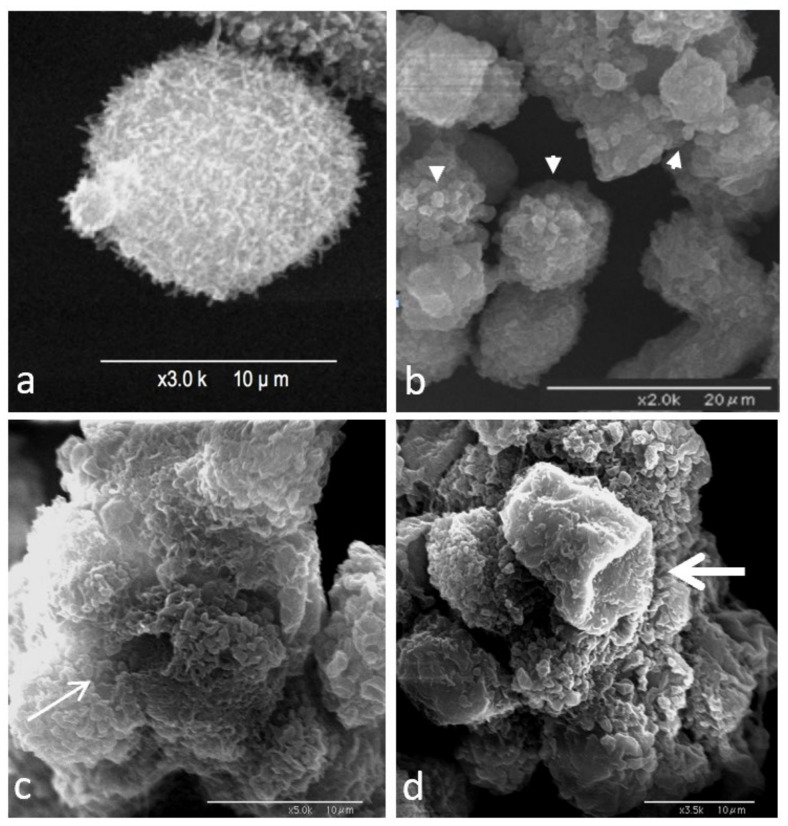
Scanning electron microscopy image of TOS-1 renal cell carcinoma. (**a**) Control TOS-1 cell in suspension covered with microvilli. (**b**) Bleb formation on the surfaces of the shock-wave-treated cells (arrowheads). (**c**) Hole formation in the cell membrane of the treated cells (thin arrow). (**d**) Collapse of the cell membrane due to damage to the underlying cell cytoskeleton (thick arrow).

Scanning electron microscopic images of the control TOS-1 cells in suspension show the cells covered with abundant microvilli on their surfaces (Figure 8a). After the shock wave exposures, however, treated cells showed various morphological deformations based on severity of the damage, such as blebs (Figure 8b), holes in the cell membrane (Figure 8c) and cell-membrane depression (Figure 8d). Bleb formation was the most common morphological finding in our experiment.

## 4. Discussion

A clinically applied extracorporeal focused shock wave is characterized by a single surge of high-pressure wave that travels through the body and exerts significant mechanical impact on the targeted area. According to numerous studies, in addition to extensive damage in the focal area, the dissipating energy of the diverging shock waves can further damage and disrupt the subcellular structures of tissues in the proximity of the targeted site [14]. Depending on the amount of energy transmitted to the cells, shock waves may induce a variety of effects, ranging from permanently damaging the cell membrane, leading to cell death, to temporarily increasing the permeability of the cell membrane, known as poration [24].

In addition, the majority of therapeutic applications of shock waves are shown to be caused by generated biological responses following antigenic enhancement of the targeted area. It is generally believed that activation of gene-signaling pathways in response to shock waves is the main mechanism responsible for antigenic enhancement [25]. However, this widely accepted theory cannot explain the mechanism behind the enhanced expression in our experiment, since the production of MSGG in TOS-1 cells is an enzymatic process and is not directly controlled by a gene pathway [26]. In addition, the time course of MSGG enhancement in our experiment suggests an immediate impact on the membrane expression of MSGG, which occurred much faster than if a gene pathway had been activated.

Nevertheless, the tumor antigens released from dead cells could have been the antigenic source of the immediately positive MSGG signals detected by flow cytometry. However, the results of double-staining flowcytometry with mAb-RM1 and propidium iodide (PI) clearly demonstrated the increased membrane expression of MSGG markers on the surfaces of the viable cells in the shock-wave-treated samples. Thus, it excludes the possibility of positive results caused by cross reaction with antigens released from dead cells or expressed on their debris. Therefore, the antigenic enhancement observed here must be the product of a mechanism other than a “gene-upregulation” pathway.

In principle, the effects of shock waves on cells are a result of a number of physical and biological factors [27]. Upon reaching the cell membrane, the microstreaming behind shock waves can generates shear stress due to differences in acoustic impedance between the lipid bilayer and the surrounding medium. The thickness of the shock front in tissue/cell medium is in the order of 1 to 2 nm (a few mean free paths of the water molecules) [28], which is less than the thickness of the cell membrane (5 to 10 nm order, including the lipid bilayer and embedded proteins) [28]. Therefore, the shock wave can directly interact with the cell membrane and temporally increase the membrane permeability [28]. The discontinuous shock can then propagate inside the cell to impinge upon the subcellular structures, e.g., nucleus, mitochondria and endoplasmic reticulum. The impulsive acceleration of the cell membrane by shock waves generates interfacial instability [29]. The coupling between the pressure gradient across the shock front and the density gradient across the membrane induces baroclinic vorticity to perturb this interface and causes the instability [30]. The re-shocking amplifies the perturbations to enhance the fluid motion across the lipid membrane. The direct impact of shock waves caused by shock wave propagation in the non-uniform structure of the cell is believed to be responsible for many biological effects of shock wave exposures.

Focused shock waves can also impact cells through cavitation, which is formed by the rarefaction wave behind the shock wave. The collapse of cavitation bubbles produces a local shock wave that can affect the nearby cells by the above-mentioned mechanisms. The bubble collapse can also produce sonoluminescence and reactive oxygen species, which further damage the targeted cells [31]. Additionally, bubble collapse can produce liquid jets near the rigid boundaries (e.g., cultured cells), which can result irreversible poration and cell death [32]. Nonetheless, liquid-jet effects were minimal in our experimental setups with the suspended cells, as discussed previously [24].

The shock waves of 16 MPa and 0.04 mJ/mm^2^ used in our experiments were in a lower range of 7–80 MPa and 0.03–1.0 mJ/mm^2^, in line with the pressure and energy flux density usually employed in extracorporeal shock wave therapy [33]. In addition, the number of shock waves applied to the cell suspension was adjusted in accordance with the focal-to-suspension volume ratio, which was comparable to the number required to induce membrane poration in the culture [14]. Furthermore, the low-negative-energy flux density of 0.002 mJ/mm^2^ indicates weak and insignificant cavitation. Accordingly, the bulk thermal effect usually observed as a result of ultrasound exposure was also small and negligible. Instead, the majority of the effects in the present experiments were produced by direct shock wave impulse and strong microstreaming (7.9 m/s on average) in the focal region.

At the cellular level, on the other hand, these physical stresses were translated into biological reactions, a process known as mechanotransduction. Shock-wave-induced mechanotransduction is believed to be responsible for many short- and long-term bioeffects that are observed in clinics. During this process, ion influx via stimulated mechanosensitive ion channels in cells under stress immediately converts the mechanical stresses exerted on the cell membrane into a cascade of intracellular biochemical events [34]. Other sources of the increased intracellular Ca^2+^ level, besides influx of extracellular Ca^2+^ as the main source of the increased ions, include release of Ca^2+^ from internal sources, such as mitochondria [35,36]. Increased ions in the cytoplasm, especially Ca^+^, can eventually promote disruption of the cortical actin filaments and perinuclear retraction of actin filaments in the shock-wave-treated cells. Actin filament disassembly plays an important role in subsequent biological events [14,37].

Bleb formation in shock-wave-treated cells, shown in Figure 8B, was the result of the breakdown of cortical actin filaments and their separation from the cell membrane. In general, blebbing is a good indicator of increased hydrostatic pressure, since its primary function is to protect the damaged cells from such an increase in hydrostatic pressure [38]. The increased intracellular calcium ion and hydrostatic pressure gradients associated with damaged cytoskeletons can in turn affect the physical properties of the cytoplasm in the shock-wave-treated cell, such as its viscosity.

Modified viscosity may further enhance intracellular flow within the cell [39]. Nevertheless, microstreaming alone cannot fully account for the dramatic change in the distribution pattern of the MSGG markers from scattered patterns in the cytoplasm into vesicle-oriented membranous structures, seen in our experiment. Instead, the modifications in physical and chemical properties of the cytoplasm of shock-wave-treated TOS-1 cells with abundant numbers of cytosolic lipid vesicles may have contributed to the formation of micelles/vesicles. In such an environment, the high concentration of cytosolic lipid-rich molecules with low surface tension, such as MSGG, may have accelerated the growth of the newly formed vesicle structures shown in Figure 6 and Figure 7.

The increased expression of the MSGG on the surface of the treated cells immediately after the shock waves, on the other hand, might be best explained by the vesicle-based cell-membrane repair response reported by McNeil and Terasaki (2001) [40]. They proposed that the membrane-repair response would allow the cells to maintain their viability despite constant mechanical damages. The membrane-repair system hypothesizes that a breach in the plasma membrane and influx of Ca^2+^ into the cytoplasm causes vesicles present in the cytoplasm under the disrupted cell-membrane site to fuse rapidly with one another or with the adjacent plasma membrane (Figure 7D). In other words, the influx causes multiple rapid exocytosis events and a simultaneous patch of lipid vesicles with the damaged site that blocks the flow into/out of the plasma membrane [41,42].

**Figure 9:**
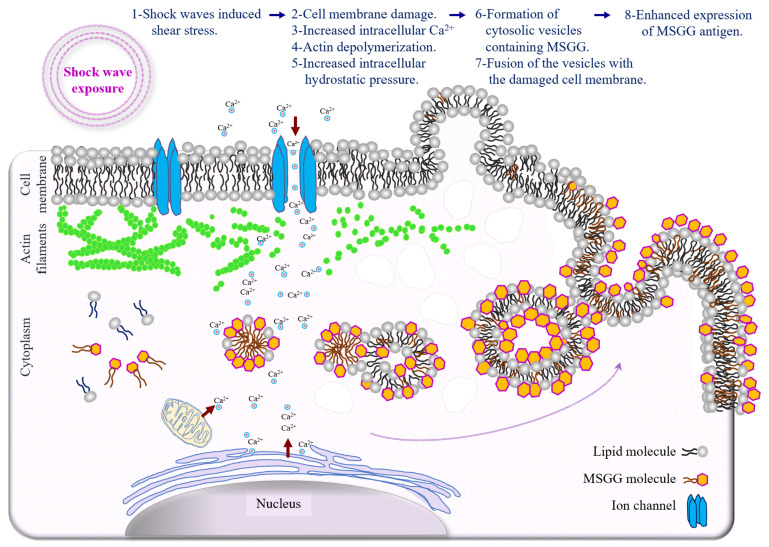
Schematic diagram depicting the mechanism of acute mechanical modification of GSL-MSGG expression on the cell membrane of renal cell carcinoma. Increased membrane tension by shear stress and shock wave interaction with intracellular structures can directly trigger a cascade of events, which begins with mechanical damage to the cell membrane, followed by an influx of extracellular Ca^2+^, damage to the cytoskeleton and increased intracellular hydrostatic pressure, which can induce cytosolic flow; lipid aggregation in the cytoplasm; vesicle formation; and incorporation of lipid molecules, including MSGG, with newly formed vesicles. Finally, fusion of the vesicles containing MSGG with the cell membrane to repair the damaged area can cause increased expression of MSGG on the cell membrane.

The type of blebs formed after shock waves can further support this theory. Generally, blebs in the cell membrane of suspended cells can either be balloon-type or blister-type [43]. The balloon-type blebs were driven from the endoplasmic reticulum and contained cytoplasmic organelles, such as small, round lipid vesicles, whereas the blister- type blebs filled with clear fluid were formed when cytoplasmic lipids integrated with the plasma membrane. The immunoelectron microscopic images of the treated cell membranes show the high level of MSGG incorporation in fluid-filled blebs containing no cytoplasmic organelles, indicating that bleb formation used cytoplasmic lipid molecules, including MSGG lipid molecules, joining the plasma membrane. Therefore, it can be concluded that resealing of the damaged membrane by the vesicle structures containing MSGG located adjacent to the plasma membrane is the source of immediately increased MSGG expression on the surfaces of the treated cells in our experiment. Continued recruitment of the lipid vesicles for the cell-membrane repair process after the initial damage might explain the peak in surface expression of MSGG around one hour after the initial impact. Figure 9 summarizes the chain of events responsible for the acute mechanical modification of MSGG expression on the cell membrane of renal cell carcinoma immediately after the shock waves.

## 5. Conclusions

Our results have demonstrated, for the first time, that shock waves can enhance the expression of intracellular tumor markers on the surface of cells through mechanisms other than the stimulation of gene pathways. In addition, we discussed the chain of events responsible for the immediate but transient antigenic enhancement on the shock-wave-treated tumor cells, whereas the value on the viable cells remained significantly higher than the control level for a considerable period of time before recovering to the normal level. An instant mechanotransduction process initiated by microstreaming shear stress is believed to have caused damages to the cell membrane and the cell cytoskeleton, which resulted in mechanochemical changes in the cytoplasm. These changes further caused formation and growth of cytosolic vesicles from cytosolic lipid molecules, including MSGG markers. The cell-membrane repair response of the stimulated cells later facilitated incorporation of the lipid vesicles into the cell membrane. As a result, the expression of the cytosolic MGSS was enhanced on the surface of the shock-wave-treated cells.

Although physical parameters are important factors in determining cellular responses following shock waves, the final results can be influenced by biological factors, such as heterogeneity of different cell types, especially at the subcellular level. Furthermore, it remains to be seen whether the molecular structure of the markers, e.g., lipid or protein, has any implications for exposing or enhancing hidden cytosolic markers by shock waves.

Physically enhancing expression of tumor antigens on the surface of cells by shock waves could be used as a new strategy to manipulate the immunogenicity of tumor cells for potential diagnostic and therapeutic purposes. In combination with other immunotherapeutic strategies, extracorporeal shock waves can also alter the tumor microenvironment and cellular structure in such a way that they become more sensitive to the cytotoxicity of currently available drugs or more immunogenic for the immune system. Further understanding in details of the impact of shock wave on tumor antigens in general and its ability to expose hidden antigen/markers in particular is essential for potential applications of shock waves in combination with cancer immunotherapy.

## Data Availability

Data are contained within the article.
